# Supplementary material for: Cleaning symbiosis in coral reefs of Jardines de la Reina National Park
Source: PeerJ. 2023 Dec 6;11:e16524. doi: 10.7717/peerj.16524 (PMC10710127; doi:10.7717/peerj.16524)
Supplement: Supplemental Information 1 — Only the categories Critically Endangered (CR), Endangered (EN) Vulnerable (VU), and Near Threatened (NT) were taken into account. [file peerj-11-16524-s001.docx]

**Cleaning symbiosis in coral reefs of Jardines de la Reina National Park**

Andy Joel Corso^1^, Fabián Pina-Amargós^2^, Leandro Rodríguez-Viera^3,^*

^1^ Center for Marine Research, University of Havana, Havana, Cuba

^2^ Blue Sanctuary-Avalon, Ciego de Ávila, Cuba.

^3^ Faculty of Marine and Environmental Sciences, Campus de Excelencia Internacional del Mar (CEIMAR), University of Cadiz, Puerto Real, Cadiz, Spain.

Corresponding Author:

Leandro Rodríguez-Viera

Email address: [leokarma@gmail.com;leandro.rodriguez@uca.es](mailto:leokarma@gmail.com;leandro.rodriguez@uca.es)

Table S1: Client species detected in reefs of Jardines de la Reina National Park, Cuba, during surveys carried out between 2019 and 2022. Classification of functional groups according to Choal *et al*. (2004), Micheli *et al.* (2014), Bonaldo *et al*. (2014), Adam *et al*. (2015), and Navarro-Martínez *et al*. (2022). The category of threat according to the IUCN. Only the categories Critically Endangered (CR), Endangered (EN) Vulnerable (VU), and Near Threatened (NT) were taken into account.

| Species | | Functional group | IUCN category |
| --- | --- | --- | --- |
| Pomacentridae | |  |  |
|  | *Abudefduf saxatilis* (Linnaeus, 1758) | Cryptic microinvertivore |  |
|  | *Chromis cyanea* (Poey, 1860) | Cryptic planktivore |  |
|  | *Chromis multilineata* (Guichenot, 1853) | Cryptic planktivore |  |
|  | *Microspathodon chrysurus* (Cuvier, 1830) | Cryptic grazer |  |
| Acanthuridae | |  |  |
|  | *Acanthurus chirurgus* (Bloch, 1787) | Roving grazer |  |
|  | *Acanthurus coeruleus* Bloch & Schneider, 1801 | Roving browsers |  |
|  | *Acanthurus tractus* Poey, 1860 | Roving grazer |  |
| Carangidae | |  |  |
|  | *Caranx ruber* (Bloch, 1793) | Midwater piscivore |  |
| Serranidae | |  |  |
|  | *Cephalopholis cruentata* (Lacepède, 1802) | Roving piscivore |  |
|  | *Cephalopholis fulva* (Linnaeus, 1758) | Roving piscivore |  |
|  | *Epinephelus guttatus* (Linnaeus, 1758) | Roving predator |  |
|  | *Epinephelus striatus* (Bloch, 1792) | Roving predator | CR |
|  | *Mycteroperca bonaci* (Poey, 1860) | Roving piscivore | NT |
|  | *Mycteroperca tigris* (Valenciennes, 1833) | Roving piscivore |  |
|  | *Mycteroperca venenosa* (Linnaeus, 1758) | Roving piscivore | NT |
| Muraenidae | |  |  |
|  | *Gymnothorax funebris* Ranzani, 1840 | Cryptic predator |  |
| Pomacanthidae | |  |  |
|  | *Holacanthus ciliaris* (Linnaeus, 1758) | Cryptic sessile invertivore |  |
|  | *Holacanthus tricolor* (Bloch, 1795) | Roving sessile invertivore |  |
| Holocentridae | |  |  |
|  | *Holocentrus rufus* (Walbaum, 1792) | Roving macroinvertivore |  |
|  | *Sargocentron vexillarium* (Poey, 1860) | Cryptic macroinvertivore |  |
| Ostraciidae | |  |  |
|  | *Lactophrys trigonus* (Linnaeus, 1758) | Roving macroinvertivore |  |
| Balistidae | |  |  |
|  | *Balistes vetula* Linnaeus, 1758 | Roving macroinvertivore | NT |
|  | *Melichthys niger* (Bloch, 1786) | Midwater planktivore |  |

Table S1 (continued): Client species detected in reefs of Jardines de la Reina National Park, Cuba, during surveys carried out between 2019 and 2022. Classification of functional groups according to Choal *et al*. (2004), Micheli *et al.* (2014), Bonaldo *et al*. (2014), Adam *et al*. (2015), and Navarro-Martínez *et al*. (2022). The category of threat according to the IUCN. Only the categories Critically Endangered (CR), Endangered (EN) Vulnerable (VU), and Near Threatened (NT) were taken into account.

| Species | | Functional group | | IUCN category |
| --- | --- | --- | --- | --- |
| Scorpaenidae | |  | |  |
|  | *Pterois* spp. | Cryptic predator | |  |
| Sphyraenidae | |  | |  |
|  | *Sphyraena barracuda* (Edwards, 1771) | Pelagic piscivore | |  |
| Labridae | |  | |  |
|  | *Clepticus parrae* (Bloch & Schneider, 1801) | Midwater planktivore | |  |
|  | *Lachnolaimus maxinus* (Walbaum, 1792) | Roving macroinvertivore | | VU |
| Scaridae (Subfamily) | | | | |
|  | *Scarus iseri* (Bloch, 1789) | | Roving grazer |  |
|  | *Scarus taeniopterus* Desmarest, 1831 | | Roving grazer |  |
|  | *Sparisoma aurofrenatum* (Valenciennes, 1840) | | Roving browser |  |
|  | *Scarus coelestinus* Valenciennes, 1840 | | Roving grazer |  |
|  | *Sparisoma atomarium* (Poey, 1861) | | Roving browser |  |
|  | *Sparisoma viride* (Bonnaterre, 1788) | | Roving grazer |  |
| Lutjanidae | | |  |  |
|  | *Ocyurus chrysurus* (Bloch, 1791) | | Midwater predator |  |
|  | *Lutjanus apodus* (Walbaum, 1792) | | Roving predator |  |
| Haemulidae | | |  |  |
|  | *Haemulon sciurus* (Shaw, 1803) | | Roving macroinvertivore |  |
|  | *Haemulon plumierii* (Lacepède, 1801) | | Roving macroinvertivore |  |
|  | *Anisotremus virginicus* (Linnaeus, 1758) | | Roving macroinvertivore |  |
| Kyphosidae | | |  |  |
|  | *Kyphosus* spp. | | Roving browser |  |

**Literature Cited**

Adam, T.C., Kelley, M., Ruttenberg, B.I. and Burkepile, D.E. (2015) Resource partitioning along multiple niche axes drives functional diversity in parrotfishes on Caribbean coral reefs. *Oecologia* 179: 1173-1185. DOI: 10.1007/s00442-015-3406-3

Bonaldo, R.M., Hoey, A.S., Bellwood, D.R. (2014) The ecosystem roles of parrotfishes on tropical reefs. *Oceanogr. Mar. Biol. Annu. Rev*. 52: 81-132. DOI: 10.1201/b17143-3

Choat, J.H., Clements, K.D., Robbins, W.D. (2004) The trophic status of herbivorous fishes on coral reefs. II. Food processing modes and trophodynamics. *Mar. Biol*. 145: 445-454. DOI: 10.1007/s00227-004-1341-7 2

Micheli, F., Mumby, P. J., Brumbaugh, D. R., Broad, K., Dahlgren, C. P., Harborne, A. R. Sanchirico, J. N. (2014) High vulnerability of ecosystem function and services to diversity loss in Caribbean coral reefs. *Biol. Conserv*., 171, 186–194. doi: 10.1016/j.biocon.2013.12.029

Navarro-Martínez, Z. M., Armenteros M., Espinosa L., Lake J.J., Apprill A. (2022) Taxonomic and functional assemblage structure of coral reef fishes from Jardines de la Reina (Caribbean Sea, Cuba). *Mar. Ecol. Prog. Ser.* 690:113-132. <https://doi.org/10.3354/meps14049>
