# Supplementary material for: Cleaning symbiosis in coral reefs of Jardines de la Reina National Park
Source: PeerJ. 2023 Dec 6;11:e16524. doi: 10.7717/peerj.16524 (PMC10710127; doi:10.7717/peerj.16524)
Supplement: Supplemental Information 2 [file MOD_1.pdf › Material_Reviewer_1/MOD_1.pdf]

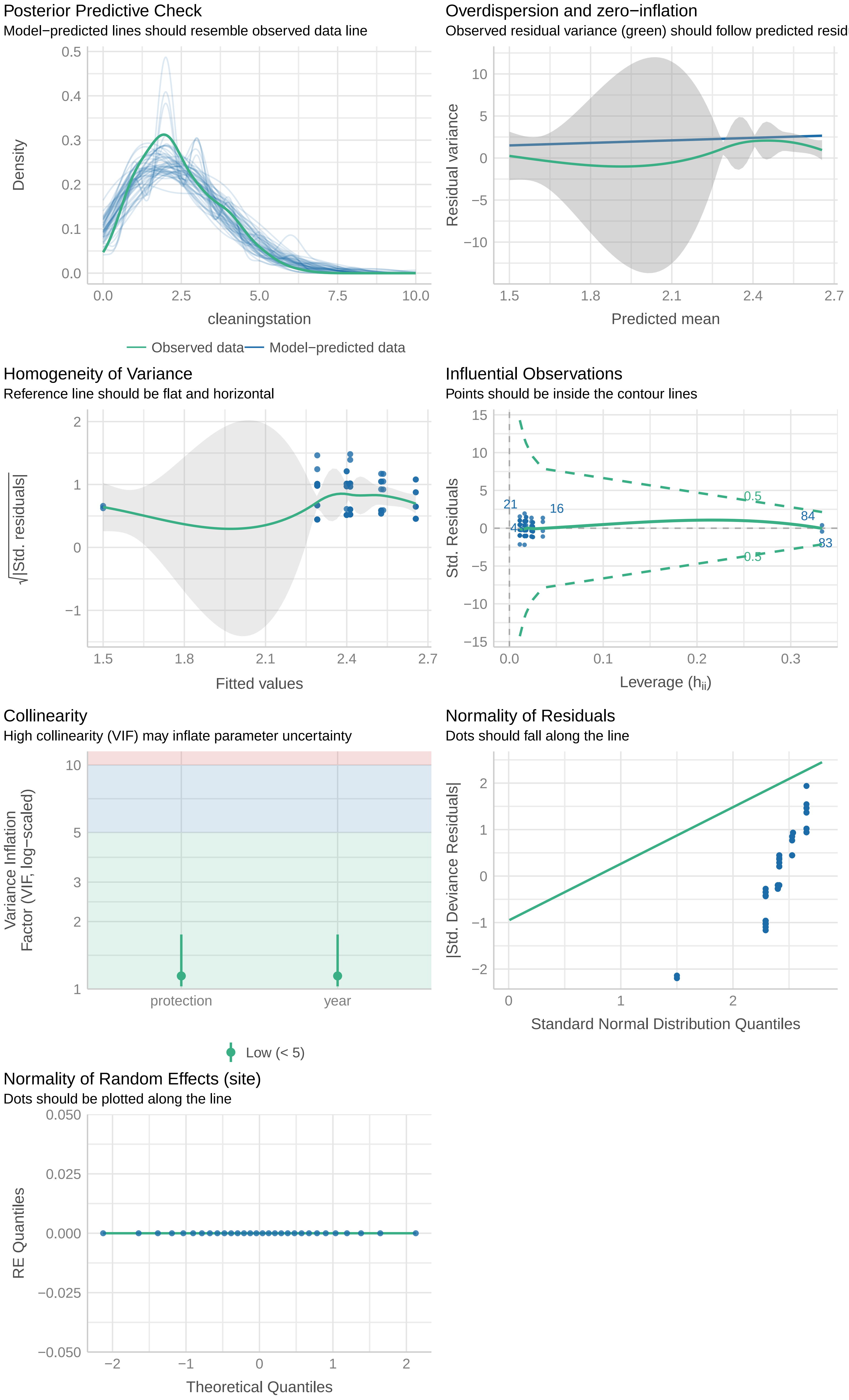
Posterior Predictive Check
Model−predicted lines should resemble observed data line
Density
cleaningstation
0.0 0.1 0.2 0.3 0.4 0.5
0.0 2.5 5.0 7.5 10.0
Observed data
Model−predicted data
Overdispersion and zero−inflation
Observed residual variance (green) should follow predicted residu
Residual variance
Predicted mean
−10 −5 0 5 10
1.5 1.8 2.1 2.4 2.7
Homogeneity of Variance
Reference line should be flat and horizontal
√|Std. residuals|
Fitted values
−1 0 1 2
1.5 1.8 2.1 2.4 2.7
Influential Observations
Points should be inside the contour lines
Std. Residuals
Leverage (hii)
−15 −10 −5 0 5 10 15
0.0 0.1 0.2 0.3
21
16
4
84
83
0.5
-0.5
Collinearity
High collinearity (VIF) may inflate parameter uncertainty
Variance Inflation Factor (VIF, log−scaled)
1 2 3 5 10
protection
year
Low (< 5)
Normality of Residuals
Dots should fall along the line
|Std. Deviance Residuals|
Standard Normal Distribution Quantiles
−2 −1 0 1 2
0 1 2
Normality of Random Effects (site)
Dots should be plotted along the line
RE Quantiles
Theoretical Quantiles
−0.050 −0.025 0.000 0.025 0.050
−2 −1 0 1 2
