# Supplementary material for: Cleaning symbiosis in coral reefs of Jardines de la Reina National Park
Source: PeerJ. 2023 Dec 6;11:e16524. doi: 10.7717/peerj.16524 (PMC10710127; doi:10.7717/peerj.16524)
Supplement: Supplemental Information 2 [file MOD_10.pdf › Material_Reviewer_1/MOD_10.pdf]

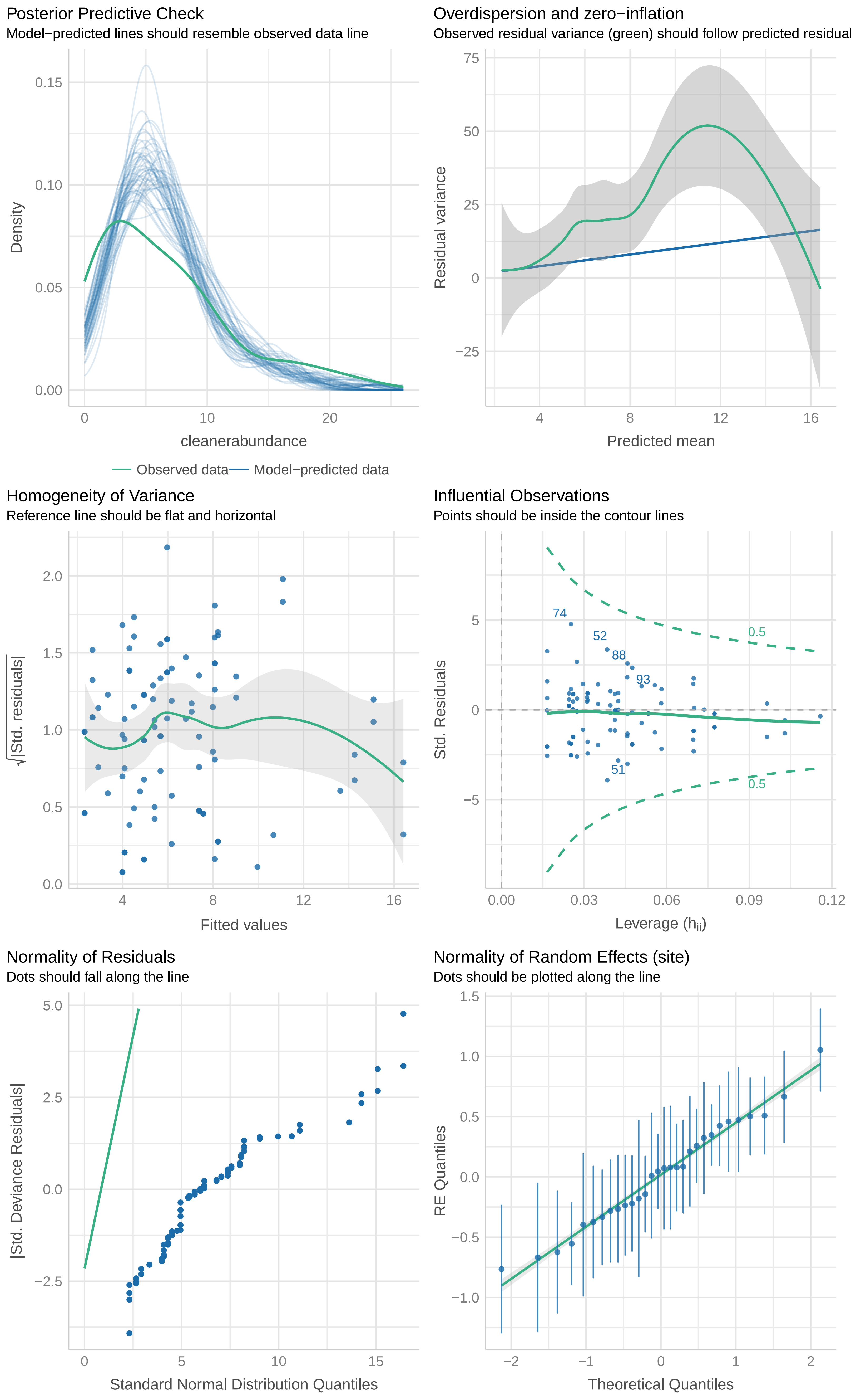
Posterior Predictive Check
Model−predicted lines should resemble observed data line
Density
0.15
0.10
0.05
0.00
0
10
20
cleanerabundance
Observed data
Model−predicted data
Overdispersion and zero−inflation
Observed residual variance (green) should follow predicted residual
Residual variance
75
50
25
0
−25
4
8
12
16
Predicted mean
Homogeneity of Variance
Reference line should be flat and horizontal
√|Std. residuals|
2.0
1.5
1.0
0.5
0.0
4
8
12
16
Fitted values
Influential Observations
Points should be inside the contour lines
Std. Residuals
5
0
−5
74
52
88
93
51
0.5
0.5
0.00
0.03
0.06
0.09
0.12
Leverage (h_{ii})
Normality of Residuals
Dots should fall along the line
|Std. Deviance Residuals|
5.0
2.5
0.0
−2.5
0
5
10
15
Standard Normal Distribution Quantiles
Normality of Random Effects (site)
Dots should be plotted along the line
RE Quantiles
1.5
1.0
0.5
0.0
−0.5
−1.0
−2
−1
0
1
2
Theoretical Quantiles
