# Supplementary material for: Cleaning symbiosis in coral reefs of Jardines de la Reina National Park
Source: PeerJ. 2023 Dec 6;11:e16524. doi: 10.7717/peerj.16524 (PMC10710127; doi:10.7717/peerj.16524)
Supplement: Supplemental Information 2 [file MOD_2.pdf › Material_Reviewer_1/MOD_2.pdf]

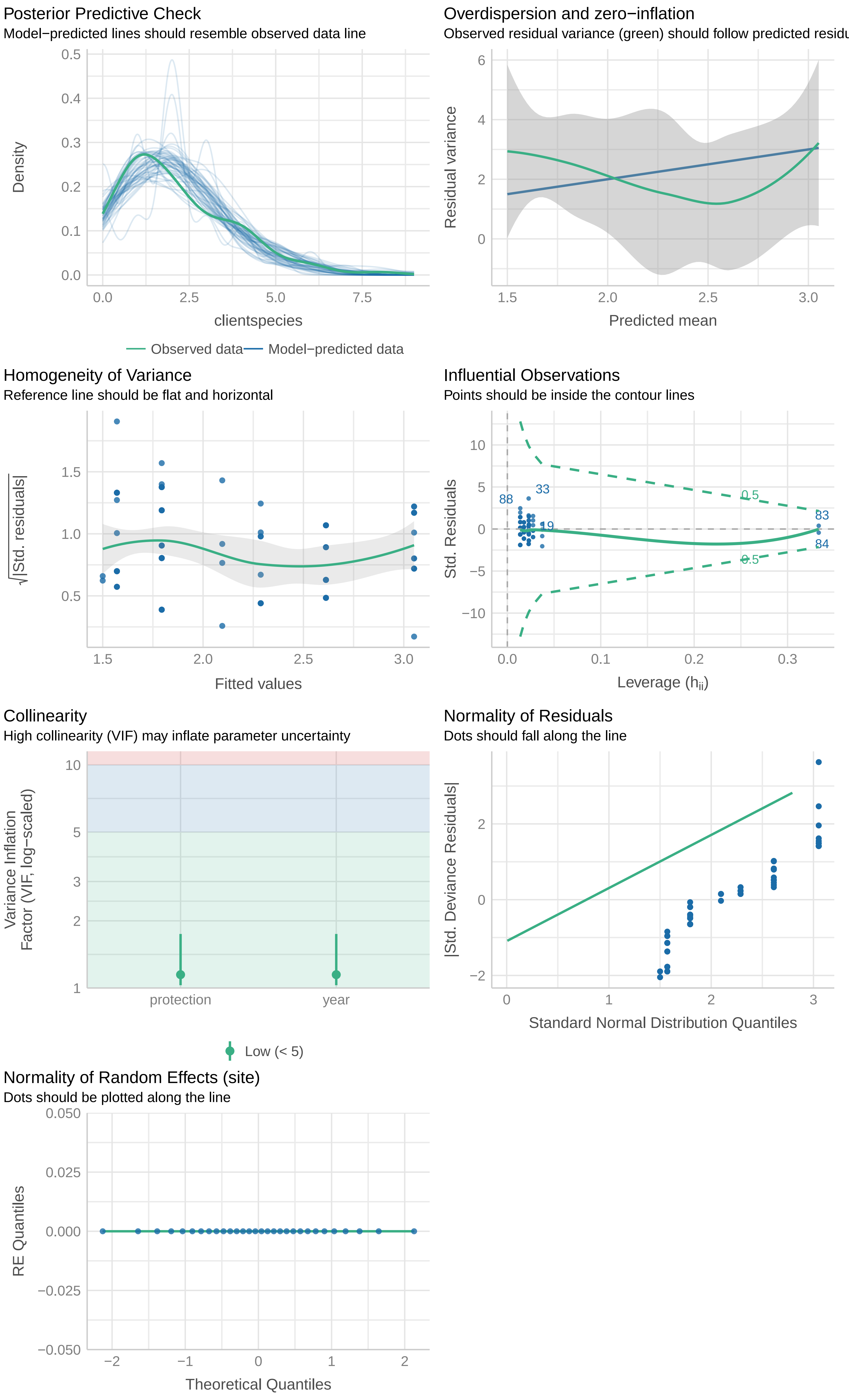
Posterior Predictive Check
Model−predicted lines should resemble observed data line
Density
clientspecies
Observed data
Model−predicted data
Overdispersion and zero−inflation
Observed residual variance (green) should follow predicted residu
Residual variance
Predicted mean
Homogeneity of Variance
Reference line should be flat and horizontal
√|Std. residuals|
Fitted values
Influential Observations
Points should be inside the contour lines
Std. Residuals
Leverage (h_{ii})
88
33
19
83
84
0.5
Collinearity
High collinearity (VIF) may inflate parameter uncertainty
Variance Inflation Factor (VIF, log−scaled)
protection
year
Low (< 5)
Normality of Residuals
Dots should fall along the line
|Std. Deviance Residuals|
Standard Normal Distribution Quantiles
Normality of Random Effects (site)
Dots should be plotted along the line
RE Quantiles
Theoretical Quantiles
