# Supplementary material for: Cleaning symbiosis in coral reefs of Jardines de la Reina National Park
Source: PeerJ. 2023 Dec 6;11:e16524. doi: 10.7717/peerj.16524 (PMC10710127; doi:10.7717/peerj.16524)
Supplement: Supplemental Information 2 [file MOD_4.pdf › Material_Reviewer_1/MOD_4.pdf]

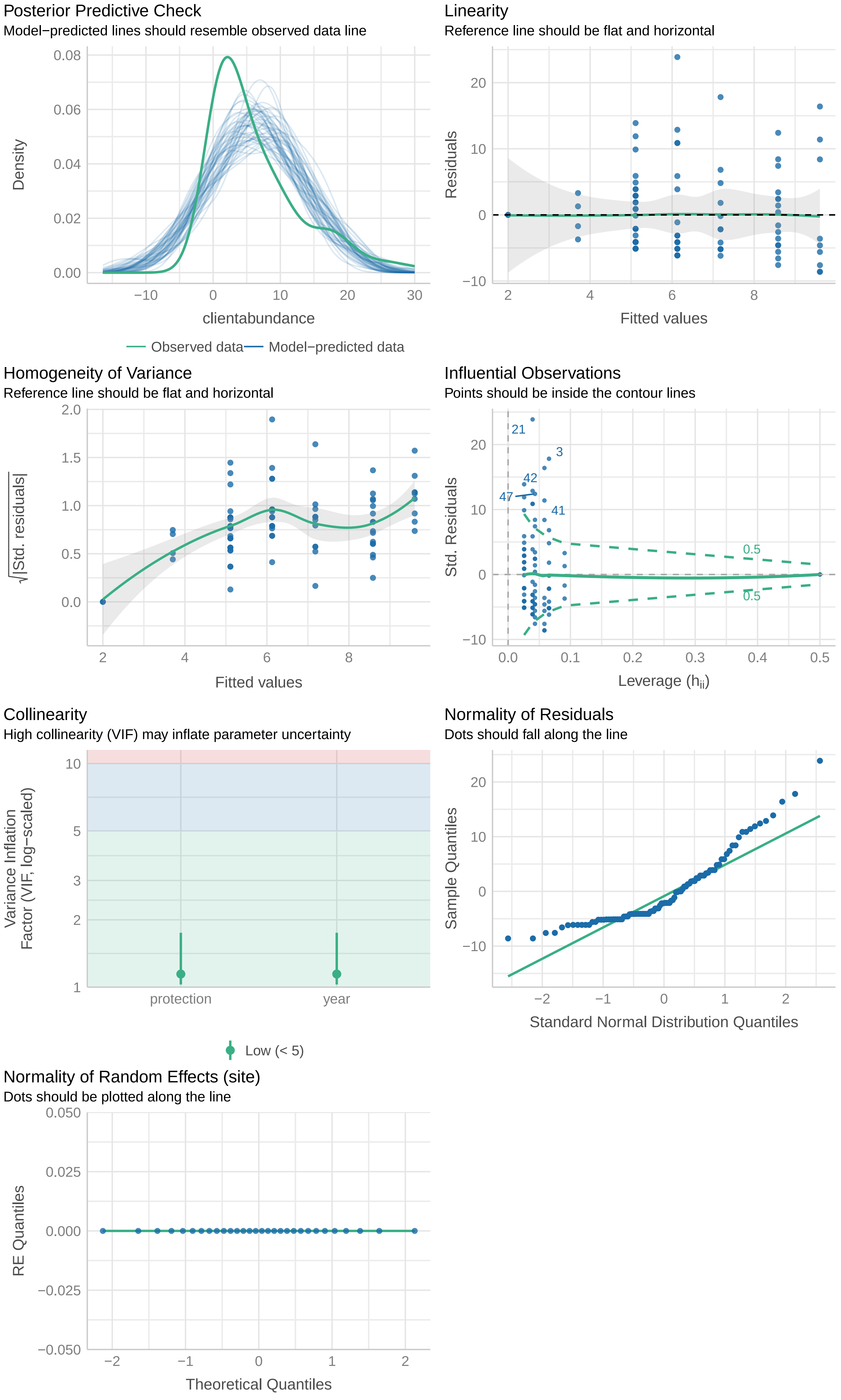
Posterior Predictive Check
Model−predicted lines should resemble observed data line
Density
0.08
0.06
0.04
0.02
0.00
−10
0
10
20
30
clientabundance
Observed data
Model−predicted data
Linearity
Reference line should be flat and horizontal
Residuals
20
10
0
−10
2
4
6
8
Fitted values
Homogeneity of Variance
Reference line should be flat and horizontal
√|Std. residuals|
2.0
1.5
1.0
0.5
0.0
2
4
6
8
Fitted values
Influential Observations
Points should be inside the contour lines
Std. Residuals
20
10
0
−10
21
3
42
47
41
0.5
0.5
0.0
0.1
0.2
0.3
0.4
0.5
Leverage (h_{ii})
Collinearity
High collinearity (VIF) may inflate parameter uncertainty
Variance Inflation Factor (VIF, log−scaled)
10
5
3
2
1
protection
year
Low (< 5)
Normality of Residuals
Dots should fall along the line
Sample Quantiles
20
10
0
−10
−2
−1
0
1
2
Standard Normal Distribution Quantiles
Normality of Random Effects (site)
Dots should be plotted along the line
RE Quantiles
0.050
0.025
0.000
−0.025
−0.050
−2
−1
0
1
2
Theoretical Quantiles
