# Supplementary material for: Cleaning symbiosis in coral reefs of Jardines de la Reina National Park
Source: PeerJ. 2023 Dec 6;11:e16524. doi: 10.7717/peerj.16524 (PMC10710127; doi:10.7717/peerj.16524)
Supplement: Supplemental Information 2 [file MOD_5.pdf › Material_Reviewer_1/MOD_5.pdf]

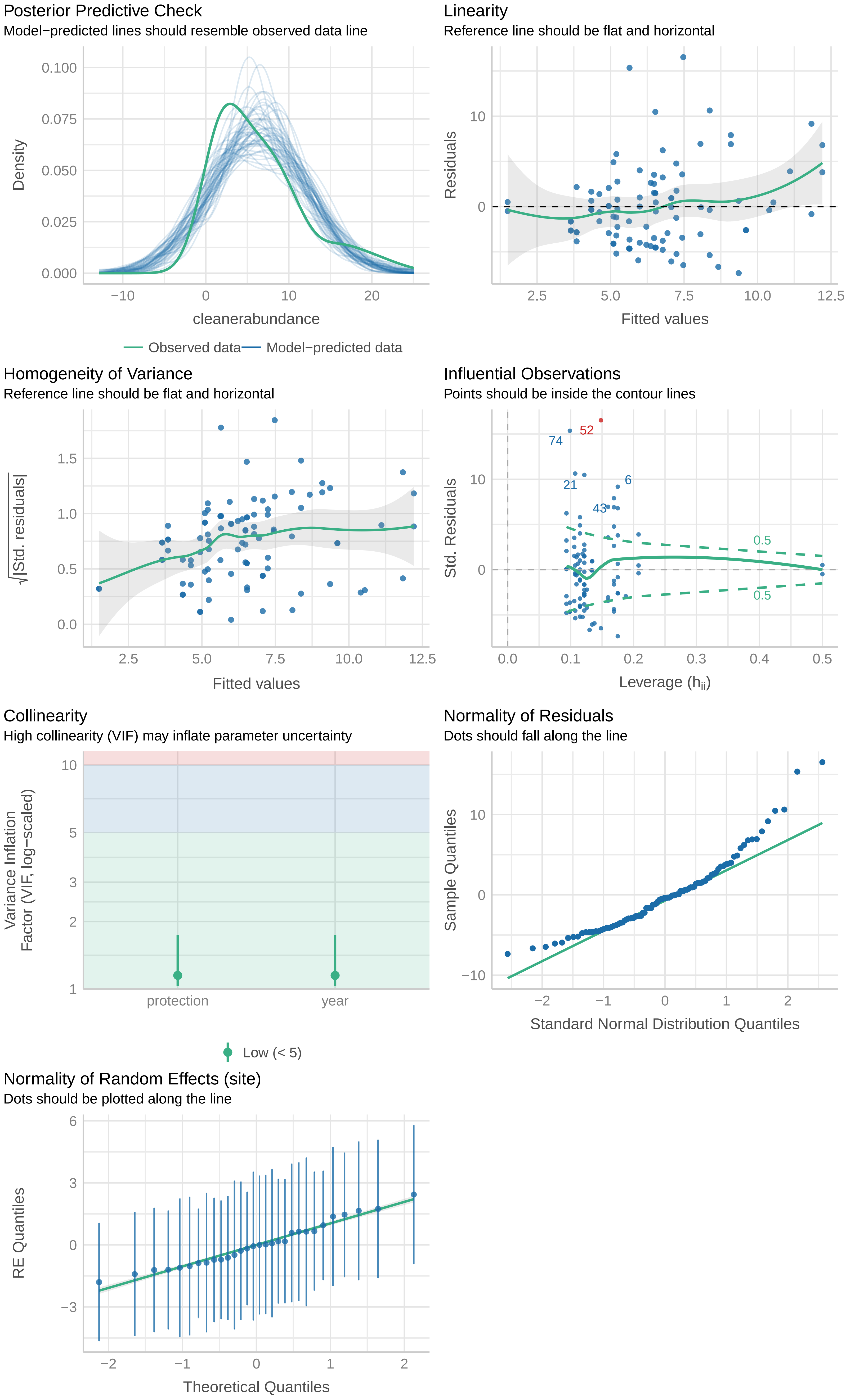
Posterior Predictive Check
Model−predicted lines should resemble observed data line
Density
cleanerabundance
0.000
0.025
0.050
0.075
0.100
−10
0
10
20
Observed data
Model−predicted data
Linearity
Reference line should be flat and horizontal
Residuals
Fitted values
2.5
5.0
7.5
10.0
12.5
Homogeneity of Variance
Reference line should be flat and horizontal
√|Std. residuals|
0.0
0.5
1.0
1.5
Fitted values
Influential Observations
Points should be inside the contour lines
Std. Residuals
Leverage (hii)
74
52
21
6
43
0.5
0.5
0.0
0.1
0.2
0.3
0.4
0.5
Collinearity
High collinearity (VIF) may inflate parameter uncertainty
Variance Inflation Factor (VIF, log−scaled)
protection
year
Low (< 5)
Normality of Residuals
Dots should fall along the line
Sample Quantiles
Standard Normal Distribution Quantiles
Normality of Random Effects (site)
Dots should be plotted along the line
RE Quantiles
Theoretical Quantiles
